# Vitamin and mineral supplementation for β-thalassemia during COVID-19 pandemic

**DOI:** 10.2144/fsoa-2020-0110

**Published:** 2020-08-18

**Authors:** Annette d'Arqom, Melvanda G Putri, Yovani Savitri, Andi Muh Rahul Alfaidin

**Affiliations:** 1 Department of Pharmacology & Therapy, Faculty of Medicine, Universitas Airlangga, Surabaya, 60131, Indonesia; 2 Faculty of Medicine, Universitas Airlangga, Surabaya, 60131, Indonesia

**Keywords:** ascorbic acid, cholecalciferol, good health and well being, immunity, SARS-CoV-2, selenium, tocopherol, zinc

## Abstract

**Aim:**

Low levels of immune-related micronutrients have been identified in β-thalassemia samples. Moreover, the excess amount of iron, contributing to oxidative stress in the pathogenesis of the disease, alters the immune system in β-thalassemia, which is important during the COVID-19 pandemic.

**Materials & Methods:**

Searches of PUBMED and EMBASE were conducted to identify the level and supplementation of micronutrients in β-thalassemia, published from 2001–May 2020.

**Results:**

The review found six observational and five interventional studies supporting the importance of supplementing vitamins and minerals among patients with β-thalassemia.

**Conclusion:**

Supplementation of immune-related vitamins and minerals might bring benefits to the immune system, especially in reducing oxidative stress in β-thalassemia.

## Lay abstract

Nutrition is important for the human body, including the immune system. During COVID-19, maintaining immunity is crucial to prevent transmission, because vaccines remain under development and definitive therapy is under clinical trial. In β-thalassemia, for which immune-related vitamins and minerals are inadequate, supplementation is important, not only to boost the immune system during this outbreak but also to maintain other body functions.

The COVID-19 outbreak, caused by severe acute respiratory syndrome coronavirus 2 (SARS-CoV-2) infection, has disseminated globally [1]. The symptoms vary from mild to severe, with severe phenotypes more prevalent in certain populations with comorbid diseases and associated with lower immune cells [2] and higher cytokine levels [3,4]. The immune system involvement in this emerging disease has been reviewed intensively [4,5]. Because vaccines remain under development and definitive therapy is under clinical trial, nonpharmaceutical interventions such as hand hygiene, social distancing and maintaining immunity are ways to protect from this infection [6]. Even though the two epicenters of COVID-19 are in China and Italy [7], which have a high prevalence of thalassemia, confirming the association between these two conditions has not been well established.

Thalassemia is the most common genetic disease with defects in α-globin or β-globin genes resulting in a reduced hemoglobin production. In its most severe form, patients with thalassemia require routine red blood cell transfusions immediately after birth to survive; thereby, causing economic, mental and social burden for patients and their countries [8]. This review focuses on β-thalassemia, which is one type of thalassemia caused by mutations in the β-globin gene leading to defective of β-globin chain synthesis. Deficiencies of micronutrients have been observed in transfusion-dependent β-thalassemia, including in immune-related vitamins and minerals, such as vitamin C, vitamin E, vitamin D, zinc and selenium [9,10]. The deficiencies might be caused by inadequate food intake, mount losses, or increasing endogenous needs for key micronutrients [10]. The low level of those micronutrients and the oxidative stress-induced by iron overload might contribute to the alteration of the immune system in β-thalassemia [8,11,12]. Therefore, this study attempted to identify the level of immune-related vitamins and minerals among patients with β-thalassemia and the necessity of the supplementations to improve immunity, which may be important during the COVID-19 pandemic.

## Methods

This review was designed and conducted based on the recommendations of PRISMA guidelines [13]. An electronic search of medical literature using the keyword (thalassemi*:ab,ti AND ‘vitamin c’:ab,ti) OR (thalassemi*:ab,ti AND ‘ascorbic acid’:ab,ti) OR (thalassemi*:ab,ti AND ‘vitamin e’:ab,ti) OR (thalassemi*:ab,ti AND ‘tocophero*’:ab,ti) OR (thalassemi*:ab,ti AND ‘vitamin d’:ab,ti) OR (thalassemi*:ab,ti AND ‘cholecalciferol’:ab,ti) OR (thalassemi*:ab,ti AND zinc:ab,ti) OR (thalassemi*:ab,ti AND selen*:ab,ti) AND (nutri*:ab,ti OR supplemen*:ab,ti) published from 2001 to May 2020 was performed on PUBMED and EMBASE. All the originals studies were retrieved and reviewed by two independent reviewers, and any dispute was discussed with the third reviewer. Observation or intervention studies reporting level of micronutrients among patients with β-thalassemia patients were included in this study. Other studies not meeting the criteria were excluded.

## Results

**Figure 1.**
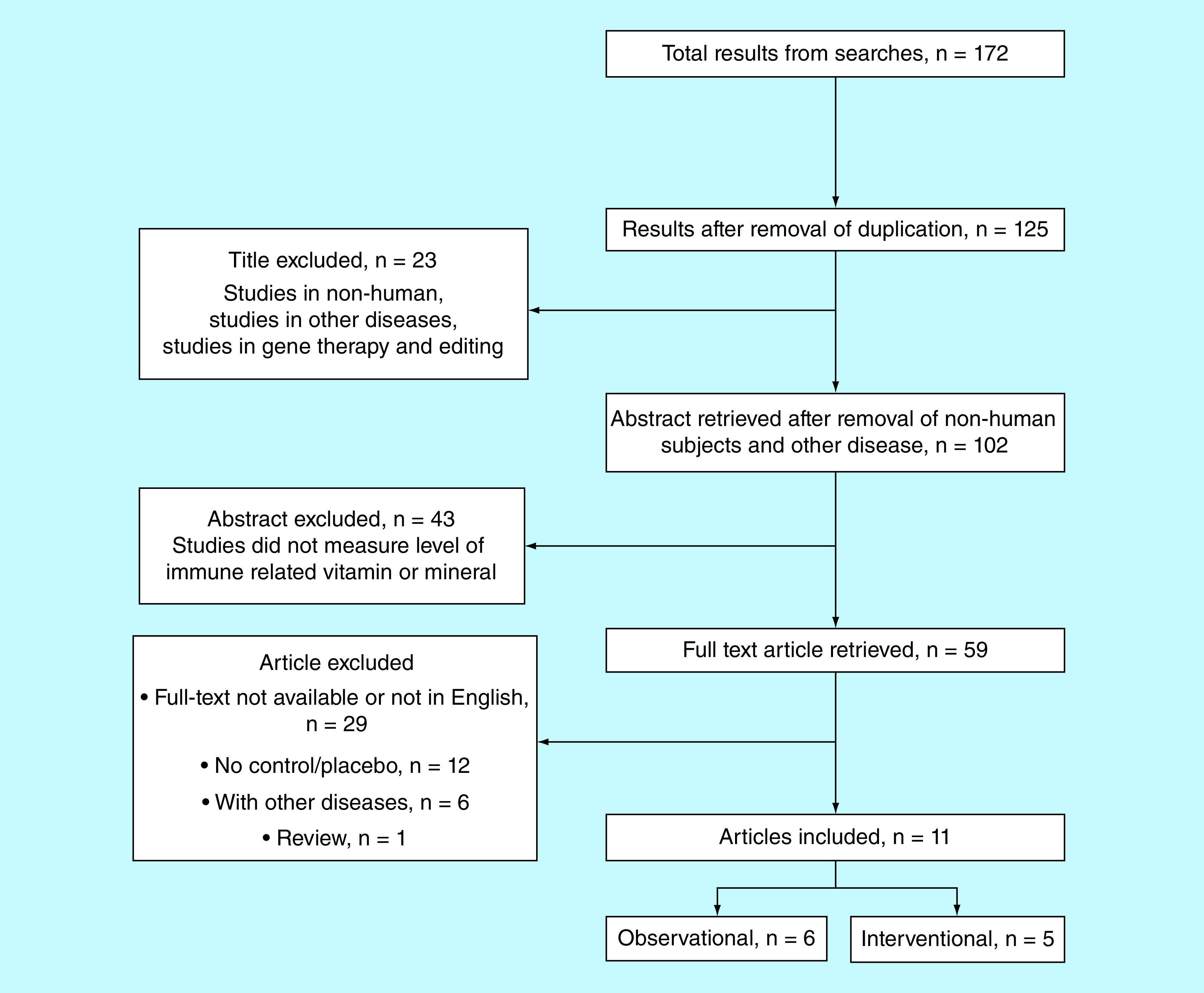
Flow diagram of articles identified and evaluated.

We identified 172 published reports of interventional and observational studies on immune-related vitamins and minerals among patients with β-thalassemia. After an initial review of the titles and abstracts, 59 studies were selected for detailed assessment, yielding six eligible observational studies and five interventional studies (Figure 1). All the patients in the observational studies were β-thalassemia major, while in the interventional studies, from 202 patients, five had HbE/β-thalassemia, 15 patients had β-thalassemia intermedia and the rest involved β-thalassemia major.

### Status of immune-related vitamin and mineral among patients with β-thalassemia

The six observational and five interventional studies, regardless of the aims of the studies, on micronutrients in β-thalassemic patients were included in this review, with 609 β-thalassemia major, 9 HbE/β-thalassemia, 15 β-thalassemia intermedia, with or without iron chelation consumption, and 248 healthy controls. The age range was from 1 to 51 years old. The immune-related micronutrient levels are summarized in Table 1, while the effects of vitamin and mineral supplementation are summarized in Table 2.

**Table 1.** Immune-related vitamin and mineral level among patients with β-thalassemia.

Vitamin/mineral	Thal subject (mean + SD)	Control (mean + SD)	Age (years)	Chelation	Ref.
Vitamin C (mg/dl) NR: 0.6–2	0.256 ± 0.09 (n = 108)	1.04 ± 0.4 (n = 60)	2–17	Deferiprone, deferoxamine or both	[9]
Vitamin E/α-Tocopherol (mg/dl) NR: 0.55–1.7	0.498 ± 0.6 (n = 108)	10.6 ± 4.5 (n = 60)	2–17	Deferiprone, deferoxamine or both	[9]
	0.2 ± 0.34 (n = 43)	1.1 ± 0.82 (n = 42)	1–15	No chelation data available	[14]
25-OHD (ng/ml) NR: >30	24.1(16.5–64.4)^†^ (n = 40)	28.1(25.3–33.4)^†^ (n = 17)	2–16	Deferiprone, deferasirox or combination with Deferoxamine	[15]
Zinc (μg/dl) NR: 70–125	17.8 ± 13 (n = 108)	103.6 ± 10.8 (n = 60)	2–17	Deferiprone, deferoxamine or both	[9]
	44.7 ± 24.2 (n = 40)	63.3 ± 30.3 (n = 30)	>2	Deferasirox	[16]
	109.8 + 68.15 (n = 30)	96.77 + 52.72 (n = 30)	1–10.6	Deferasirox and deferoxamine	[18]
Selenium (μg/l) NR: 70–150	31.5 ± 19.1 (n = 108)	65.9 ± 6.3 (n = 60)	2–17	Deferiprone, deferoxamine or both	[9]
	1.4 + 0.2 (n = 20)	1.8 + 0.1 (n = 10)	19–32	Deferiprone or deferoxamine	[17]

Controls are healthy subject.

† Median (range).

NR: Normal range.

**Table 2.** Supplementation of immune-related vitamin and mineral level among patients with β-thalassemia.

Vitamin/mineral	Supplement	Before (mean + SD)	After (mean + SD)	Age (years)	Outcomes	Ref.
Vitamin C (mg/dl)	100 mg vitamin C/day, 1 years	3.80 + 1.67 (n = 90)	6.40 + 1.14	≤18	Vitamin C potentiates the efficacy of DFO to reduce iron overload	[19]
Vitamin E/α-Tocopherol (mg/dl)	350 mg/day vitamin E, 1 month	0.59 + 0.41 (n = 5)^†^	5.19 + 1.37	28.5 + 6.2	Vitamin E prevent erythrocyte membrane damage	[22]
	400 mg/day vitamin E	0.3 + 0.2 (n = 30)	0.9 + 0.5	>18	Mean GPX activity, but not SOD, decreased	[20]
	2 × 300 mg/day of vitamin E, 9 months	0.27 + 0.05^§^ (n = 15)^‡^	0.79 + 0.13^§^	10–51	Vitamin E improves the antioxidant/oxidant balance in plasma, LDL particles and red blood cells, and counteracts lipid peroxidation processes	[21]
Zinc (μg/dl)	30 mg/day zinc sulfate, 9 months	68.9 + 25.5 (n = 32)	93 ± 29.8	8–18	Potential antioxidant and anti-inflammatory effects of zinc supplements in reducing anti-HSP27 titers	[23]
	Zinc sulfate 220 mg/day (Zinc 50 mg)	84.6 + 14.8 (n = 30)	163.7 + 14.5	>18	Mean GPX activity, but not SOD, decreased	[20]

† HbE/β-thalassemia.

‡ β-thalassemia intermedia.

§ Converted from μMolar.

GPX: Glutathione peroxidase; LDL: Low-density lipoprotein; SOD: Superoxide dismutase.

The immune-related vitamins and minerals, including vitamin C [9], vitamin E [9,14], vitamin D [15], zinc [9,16] and selenium [9,17], were identified to be reduced among patients with β-thalassemia when compared with healthy controls. Only one study by El Missiry *et al.* reported normal levels of zinc among 30 patients, which was comparable with healthy control [18]. Interestingly, the mean level of 25-OHD_3_ in β-thalassemia and healthy controls was lower than the sufficient reference value [15]. A similar finding was observed regarding selenium [9,17] and in zinc [16] indicating that these reduced vitamins and minerals might not only be affected by the disease status or severity.

All the patients in the observation studies consumed iron chelation agents, including deferiprone, deferasirox, deferoxamine or combination to remove the excess iron. Unfortunately, due to the characteristics of the respondents and the study design, only five interventional studies on vitamin C [19], vitamin E [20–22] and zinc [20,23] supplementation were included in this study. The details of each vitamin and mineral will be discussed in each section.

### Vitamin C

During this pandemic, vitamin C is routinely given to patients. In many countries, a high dose of vitamin C is administered intravenously to reduce the cytokine storm among patients with Acute Respiratory Distress Syndrome. Its antioxidant capacity and antiviral properties were claimed to give a good outcome among patients with COVID-19 [24] even though clinical trials of 24 gm/day of vitamin C treatment for 7 days remain under investigation [25]. *In vivo* study demonstrated that supplementing vitamin C for 3 weeks before H3N2 infection, but not on the day of infection, supports viral elimination; suggesting the importance of maintaining vitamin C level for immunity against the virus [26]. Among 84 patients with sepsis and Acute Respiratory Distress Syndrome, 4 × 50 mg/kg vitamin C for 4 days did not significantly reduce the organ failure, inflammation (C-reactive protein) and vascular injury (thrombomodulin), when compared with patients receiving placebo; however, overall, vitamin C supplementation reduced the mortality rate [27]. Nevertheless, the contrary result might be caused by the stage of sepsis in the study, the vitamin C dose and eliminating the deceased patients from the calculation [28]. In β-thalassemia, limited information is available regarding ability of vitamin C to disrupt the integrity of low-density lipoprotein, scavenge-free radicals, inhibit lipid peroxidation and restore other antioxidants such as α-tocopherol [29]. *In vitro* studies demonstrated that supplementing vitamin C and selenium can restore the cell target lysis activity of natural killer (NK) cells from non-splenectomized patients with β-thalassemia [30].

Iron is the main culprit of oxidative stress in β-thalassemia. A Fenton reaction, a reaction between iron and hydrogen peroxide, produces reactive oxygen species (ROS) and leads to tissue and organ damage contributing to the disease pathology [31]. A reduced total antioxidant capacity among patients with thalassemia was seen in a study from 165 transfusion-dependent patients with β-thalassemia [32]. In addition, decreased activity of antioxidant enzymes such as superoxide dismutase (SOD), glutathione peroxidase (GPX) and catalase might contribute to the increasing level of oxidation [20]. Therefore, an antioxidant agent, such as vitamin C or vitamin E is necessary to reduce oxidative damage.

One related study also observed reduced vitamin C levels among transfusion-dependent patients with β-thalassemia when compared with healthy control [9]. Moreover, the status of oxidative stress induced by iron overload correlated with poor growth among children with β-thalassemia, suggesting the necessity of antioxidant supplementation [14]. Interestingly, in the absence of an iron chelator, vitamin C can increase iron absorption by reducing Fe^3+^ to Fe^2+^, contributes to hydroxyl radical formation and further increases oxidation. Therefore, with its prooxidant activity, vitamin C supplementation in iron-rich conditions is still debatable [33]. Despite its prooxidant activity, vitamin C increases the efficiency of chelation agents by promoting Fe^2+^ and increases the iron release from reticuloendothelial system. Therefore, in the presence of chelation agents, free iron is accessible and increases the excretion of minerals [33,34].

A clinical study investigating the effect of vitamin C on iron chelator agents was conducted among 180 transfusion-dependent patients with β-thalassemia. Patients were divided in six groups, each receiving iron chelation deferoxamine, deferiprone and deferasirox with or without vitamin C 100 mg daily. Follow up was performed for 1 year to assess transfusion interval, hemoglobin level, iron profile and iron deposition in various organs. The results showed reduced serum ferritin, liver iron concentration and transferrin index, as well as an increase in hemoglobin level and cardiac MRI T2* in the vitamin C supplemented group. Interestingly, vitamin C combined with deferoxamine increased hemoglobin level, reduced serum ferritin and transferrin saturation compared with those receiving deferiprone or deferasirox with vitamin C [19]. Therefore, supplementing vitamin C might potentiate the efficacy of deferoxamine better than deferiprone and deferasirox to bind the iron and effectively reduce the iron overload, contributing to alteration of the immune system.

In summary, considering the anti and prooxidant properties of vitamin C, its supplementation in transfusion-dependent β-thalassemia should only be considered while consuming deferoxamine with a dose of 2 to 3 mg/kg/day [35]. This dose is far higher than the recommended dietary allowance (RDA) of 90 mg/day for adult males and 75 mg/day for adult females to reach the antioxidant effects [36]. However, cautions should be taken when consuming a high dose of vitamin C as its metabolite, oxalic acid, may crystallize to calcium oxalate, which can lead to oxalate nephropathy [37].

### Vitamin E

The level of another antioxidant vitamin, vitamin E or tocopherols, also decreased in β-thalassemia major [9,14]. A study in 30 patients with β-thalassemia major found reduced vitamin E level and this level increased after 400 mg/day vitamin E supplementation, opposite that of the mean of glutathione peroxidase (GPX) activity [20]. Moreover, an oral treatment of 600 mg/day of vitamin E reduced the fragility of red blood cells among 5 patients with HbE/β-thalassemia. Unfortunately, no changes in hemoglobin levels could be observed after the supplementation [22]. The role of vitamin E supplementation in preventing erythrocyte membrane damage and increasing antioxidant activity was also observed among 15 patients with intermediate β-thalassemia receiving 350 mg/day of vitamin E [21]. The RDA for both adult males and females is 15 mg/day of vitamin E (α-tocopherol) [36].

Nevertheless, the antioxidant activity of vitamin E has been studied extensively. To prevent lipid peroxidation, this vitamin works by donating a hydrogen atom to the free radicals and produces oxidized tocopherol radicals. Further, with the help of vitamin C, oxidized tocopherol radicals are converted back into tocopherols [29]. Moreover, one proteomics study suggested that vitamin E might alter C3 complement expression leading to stabilizing the RBC membrane in circulation [38]. Unfortunately, a study conducted in NK cells of patients with thalassemic found that vitamin E failed to restore the cytolytic capacity of the cells [39]. However, a recent study showed that vitamin E supplementation significantly increased the risk of prostate cancer among healthy males. The risk was increased by 17% at 7 years of median follow-up [40]. *In vivo* study indicated that vitamin E upregulated CYPs, including CYP1A1 and CYP1B1 which highly expressed in prostate cancer, increased ROS production and led to the mutation of prostate cells [41]. Therefore, males with prostate hyperplasia, elevated prostate-specific antigen or history of prostate cancer should be cautious when consuming high dose of vitamin E [42].

In the clinical setting, supplementing high dose antioxidant vitamins remains a very common practice. To understand whether this supplementation has a beneficial or detrimental effect among patients with β-thalassemia, further studies involving more patients with thalassemic are needed to explore the effect of vitamin C and vitamin E supplementation in the pathogenesis of β-thalassemia. However, to obtain the antiviral properties and maintain the immune system, patients with β-thalassemia must consume these antioxidant vitamins at the correct time and suitable dose to minimize unwanted effect.

### Vitamin D

Vitamin D is a fat-soluble sterol derivative important for bone health and plays a crucial role in calcium and phosphate homeostasis, glucose, mineral regulation and cardiovascular, neurocognitive and immune functions. The idea that vitamin D_3_ serves a role in immunity has been established because of abundant VDR expression on innate and adaptive immune cells which has been elaborated in other reviews [43–45]. The antiviral property of vitamin D has been reported in rhinovirus [46,47], hepatitis C virus [48] and influenza virus [49,50]. However, the benefit of vitamin D supplementation in respiratory infection remains debatable [51,52]. In COVID-19 infection, a positive correlation was found between the low level of vitamin D and the number of COVID-19 mortality cases [53]. However, its benefit remains questionable [54] and the confirmation is currently under investigation in some clinical trials [55–57].

Serum level of 25 hydroxycholecalciferol (25-OHD) is used to determine the vitamin D status of the patient [58,59], and is categorized as sufficient when serum level of 25-OHD was >30 ng/ml (>75 nmol/l), insufficient when serum level of 25-OHD was 20–30 ng/ml (50–75 nmol/l) and moderate deficiency when the value was <20 ng/ml (<50 nmol/l) [60]. A study in Egypt involved 40 patients with β-thalassemia and 17 healthy controls showed the mean serum level of 25-OHD of all respondents was below >30 ng/ml. Nine of 40 patients (22.5%) presented vitamin D deficiency [15]. The risk for developing vitamin D deficiency among patients with thalassemia includes liver iron deposition, darker skin, inadequate dietary and supplement intake, less sun exposure or less physical activity due to the disease burden [61]. Iron deposition in the liver might disrupt the hydroxylation of vitamin D in 25-OHD before the next hydroxylation in the kidneys in 1,25 OH_2_D_3_. Moreover, its deposition in the skin might affect vitamin D level due to disrupting vitamin D synthesis [62].

Even though altered immune function has been reported in β-thalassemia [63], such as neutrophils and lymphocytes activation, its correlation with vitamin D level has not been elucidated. The fact that transfusion-dependent thalassemia was more prone to bacterial infection supports the alteration of immune function in this genetic disease [64]. The correlation of vitamin D deficiency in β-thalassemia has been known to alter the cardiovascular system [62,65], endocrines [66] and the bone [15,67]. A study involving all types of thalassemia, including β-thalassemia, supplementation of 50,000 IU of ergocalciferol (vitamin D_2_) could increase the 25-OHD level; however, the effect of this supplementation on the immune system was not investigated [61].

Based on the evidence, the recommendations for vitamin D supplementation among patients with thalassemia treated for 8 weeks are 50,000 IU of vitamin D_2_ weekly or 2000 IU of vitamin D_3_ for the patients with serum level of 25-OHD <20 ng/ml (<50 nmol/l). For maintenance, oral intake of 800 to 1000 units vitamin D daily or 50,000 IU monthly or a mega-dose of vitamin D (10,000 IU/kg, maximum 600,000 IU) every 6 months (either orally or intramuscularly), especially for those not receiving adequate sun exposure, are recommended. Patients with serum level of 25-OHD >20 ng/ml (>50 nmol/l) can be given orally 800 to 1000 IU of vitamin D_3_ daily or 50,000 IU of vitamin D_2_ orally monthly or a mega-dose of vitamin D (10,000 IU/kg, maximum 600,000 IU) orally or IM every six months as maintenance therapy [68]. This recommendation is higher than the RDA for 1 to 70 years for men and women which is 600 IU/day or 800 IU/day for those who older than 70 years [69]. Even though the aim of this recommendation is to maintain bone health, but keeping an adequate vitamin D level might bring benefits to fight against bacterial and viral infection among patients with β-thalassemia by its modulation of the immune system.

### Zinc

Zinc is a structural element of many proteins, including zinc finger proteins, enzymes and a trace element which is important in synthesizing cholesterol reducing fat and supporting the immune and antioxidant systems [70]. It plays an essential role in bone homeostasis and bone growth as well as in maintaining healthy connective tissues [71]. This mineral is essential for growth hormone maintenance, nucleic acid synthesis, macronutrient metabolism, cell division and IGF regulation in the body [72]. Therefore, zinc deficiency might affect children's growth and development. Zinc is also associated with red blood cell survival and maintaining the integrity of the immune system; thus, a low concentration of zinc may lead to RBC membrane fragility [73] and affect immune functions such as impairing innate [74,75] and adaptive immunity [76]. Some countries have reported that zinc deficiency led to growth retardation, hypogonadism and increased mortality and morbidity from infection-related diarrhea and pneumonia because of immunocompromised conditions [77,78]. Moreover, antiviral properties of zinc also have been established such as its role in reducing the incidence [79] and duration [80] of the common cold and preventing H1N1 virus infection [81]. Even though the effects of zinc in COVID-19 remain under investigation [82,83], many advantages exist in maintaining sufficient levels of this micronutrient including in β-thalassemia.

Zinc serum level in β-thalassemia varies between normal [18] to low [9,16,23]. This variation might be caused by patients’ background before the study. Zinc deficiency among patients with thalassemia may be related to an inadequate intake of food containing zinc, kidney dysfunction or abnormality in urinary absorption of zinc, disturbance in zinc metabolism, a higher level of zinc excretion in urine and sweat and increasing oxidative stress due to hemolysis or iron chelators [84–86]. Moreover, zinc and iron are absorbed in the same intestinal mucosal cells and use transferrin as a transporter. When the iron:zinc ratio is more than 2:1, transferrin is less likely available for zinc, which disturbs zinc absorption. On top of that, increasing iron absorption from the intestinal mucosal cells of patients with thalassemia has been reported. The elevated level of iron absorption might contribute to inhibiting zinc absorption [85]. The iron chelation agents, especially deferiprone, binds the divalent zinc cations and increases zinc excretion [87]. Therefore, a 4-h interval between deferiprone and zinc supplementation is needed to prevent zinc deficiency [88].

The preferred amount of zinc supplementation in thalassemia is 45 mg daily, much higher than RDA 11 mg for adult males and 8 mg for females [89], as the antioxidant effect of 50 mg zinc supplementation successfully decreased GPX and might contribute to reducing the damage caused by free globin precipitation and iron overload [20]. However, a similar result also has been observed in the lower concentration of zinc sulfate supplementation (30 mg zinc sulfate for 9 months). Even though increasing zinc level only reached one half of that in the related study, the antioxidant effect of zinc still can be observed by the downregulating anti-HSP27 titers [23]. Albeit a clinical trial regarding the effect of zinc on cellular immunity of thalassemia major has been performed in 2017, the result has yet to be reported [90].

### Selenium

Selenium works by incorporating the protein as a selenoprotein and carries large functions including antioxidants, antivirus and anti-inflammatory [91]. Similar to zinc, selenium deficiency also affects innate and adaptive immunity and has been reviewed extensively since decades ago [92,93]. Supplementation of selenium is known to restore those immune cells in function and number. This micronutrient is also found to play roles in viral infection protection due to altering monocytes/macrophages cytokines production and CD4^+^ T cells proliferation [94,95]. The association between selenium and COVID-19 has been reported solely based on the cure rate and regional selenium status [96]. Even though the hypothesis of selenium might prove beneficial in preventing SARS-CoV-2, further investigation is necessary before its clinical application [97].

Due to iron chelator consumption, micronutrients such as selenium might be excreted from patients with β-thalassemia major and result in reduce concentration in the blood [9,17]. Unfortunately, limited studies are available on the effect of selenium in β-thalassemia, including in the immune system. Nevertheless, because selenium is a known antioxidant, any reduction of this trace element in β-thalassemia might reduce the activity of the antioxidant enzymes and leading to increased oxidative stress [98].

Because selenium is a micronutrient needed in a small amount, a thin boundary exists between the beneficial and detrimental effects of this mineral [91]. For example, a sufficient level of selenium correlates with a better immune system, reproduction, cardiovascular system and bone health. However, a high level of this micronutrient correlates with type 2 diabetes [99]. Therefore, finding the optimum dose of selenium supplementation is important, including in β-thalassemia which presents a lower selenium level. The average requirement of 45 μg/l selenium in plasma can be obtained by supplementing selenium 55 μg/day among people aged 19–50 years, 40 μg/day among people aged 9–18 years and 30 μg/day among people aged 4–8 years, with the upper limit of selenium intake set at 400 μg/day [36]. Consequently, the supplement dose for patients with β-thalassemia should be adjusted based on the patients’ selenium level and the clinical symptoms.

## Discussion & future perspective

A study between β-thalassemia and SARS-CoV-2 infection or COVID-19 has yet to be well established. A study in Italy identified 11 patients with β-thalassemia and underlying COVID-19 disease, where no increasing severity was observed [100]. Moreover, using a regression model to find a correlation between COVID-19 confirmed and mortality cases to thalassemia incidence in three different Italian regions, the study showed a negative correlation between COVID-19 and β-thalassemia incidence [101]. Regardless of the small number of respondents and short time of observation, this phenomenon might be caused by self-awareness due to self-isolation [102]; however, it would be interesting to further investigate *in vitro* and *in vivo* conditions to understand the link between β-thalassemia and COVID-19 which might bring new insight regarding the disease pathology.

Altered immunity in β-thalassemia has been reported, including a higher number in leukocytes, neutrophils and lymphocytes, lower neutrophils maturation and function due to downregulated PU.1 expression [103], impaired NK cells activity [30], phagocytic activity of monocytes and macrophages [104,105], increased numbers of CD8^+^ T cells and reduced CD4^+^ T cells among patients [106]. Despite an increasing number of immune cells, the premature aging of the lymphocytes or immunosenescence impaired the proliferative capacity and increased the expression of Fas receptor. This phenomenon might be caused by iron overload-induced oxidative stress leading to the genotoxicity of the immune cells [11].

**Figure 2.**
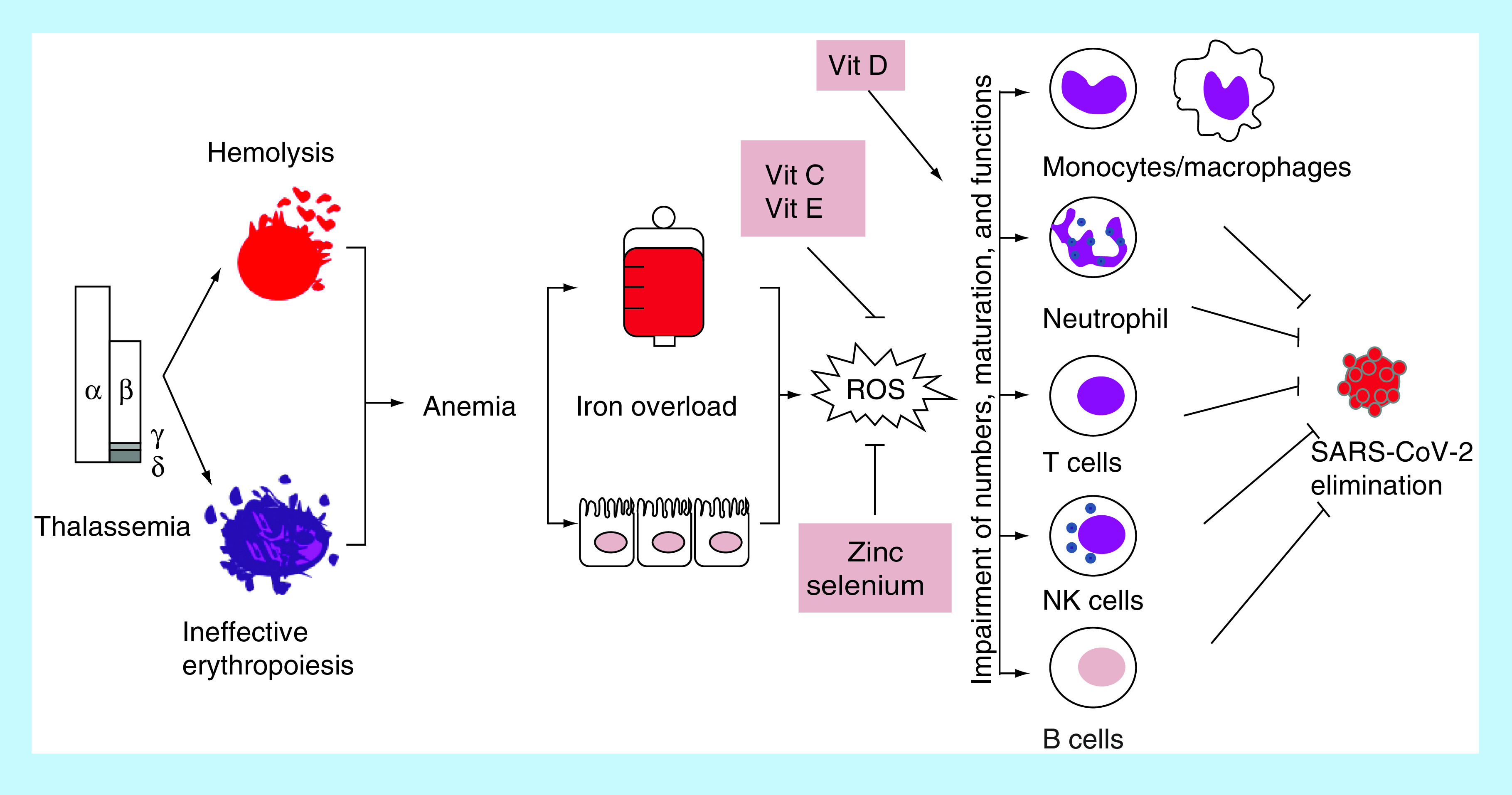
Pathophysiology of β-thalassemia and point of action of vitamins and minerals in β-thalassemia. Reduction of β-globin resulted in free α-globin for which its precipitation causes damage to cell membranes leading to hemolysis and ineffective erythropoiesis. These two conditions reduce the hemoglobin level leading to anemia. Due to routine blood transfusion and increasing iron absorption from the digestive tract, the iron level is increased and the Fenton reaction, producing reactive oxygen species occurs and alters the immune system such as impairing the proliferation, differentiation, maturation and gene expression of innate and adaptive immune cells. The defect of the immune system reduces the ability to eliminate SARS-CoV-2 and leads to further pathology of COVID-19. Vitamin C, vitamin E, vitamin D, zinc and selenium supplementations might bring advantages for immunity in β-thalassemia by reducing reactive oxygen species and improving proliferation, differentiation, maturation and gene expression of innate and adaptive immune cells. Thus, strong immunity eliminates SARS-CoV-2 effectively. NK: Natural killer; ROS: Reactive oxygen species.

Moreover, several micronutrients that decreased in thalassemia are hypothesized to be beneficial in preventing outcomes of COVID-19 (Figure 2). Because no established guidelines are available regarding the susceptibility or the immunity of patients with β-thalassemia to SARS-CoV-2 infection, prevention is crucial. Therefore, vitamin and mineral supplementation that might provide benefits for patients with β-thalassemia warrants investigation, especially in transfusion-dependent cases not only to combat the SARS-CoV-2 but also for homeostasis of other functions. However, some limitations of the current studies should be acknowledged including the lack of related research studies, the number of patients and research methods.

## Conclusion

Low levels of immune-related vitamins and minerals in β-thalassemia can be improved with supplementation. The supplementation and adequate level of micronutrients may be beneficial to the immune system, especially in reducing oxidation as a result of disease pathogenesis and iron overload from the therapy. With vaccines and effective drugs under development, maintaining immunity is important for transfusion-dependent patients with β-thalassemia during the COVID-19 pandemic.

**Executive summary**

- An inadequate level of immune-related vitamins and minerals in β-thalassemia was explored, including vitamin C, vitamin E, vitamin D, zinc and selenium supplementation.
- Vitamins and minerals supplementation is important to increase the level of micronutrients.
- The antioxidant function of micronutrients helps to reduce the oxidation as a result of disease pathogenesis and iron overload from the therapy.
- The recommended dose of the vitamins and minerals supplementation usually higher than recommended dietary allowance; therefore, patients with β-thalassemia must consume this supplementation at the correct time and suitable dose to minimize the unwanted effects.
- The interval between providing iron chelator agents and supplements should be considered to maximize the beneficial and reduce the negative effects of both agents.
- Maintaining immunity is important for transfusion-dependent patients with β-thalassemia during the COVID-19 pandemic which can be achieved with supplementation of immune-related vitamins and minerals.
